# *Artemisia argyi* Polysaccharides Protect Intestinal Health by Enhancing Antioxidant Capacity in Suckling Lambs Under Cold Exposure

**DOI:** 10.3390/ani16162512

**Published:** 2026-08-12

**Authors:** Shuang Zheng, Xiao Jin, Jian Song, Xintong Li, Zhipeng Han, Bo Wang, Dengsheng Sun

**Affiliations:** 1 College of Animal Science, Inner Mongolia Agricultural University, Hohhot 010018, China; 13284928933@163.com (S.Z.); 18031478235@163.com (J.S.); lixintongaa@163.com (X.L.); hzp4639@163.com (Z.H.); 2 State Key Laboratory of Animal Nutrition and Feeding, College of Animal Science and Technology, China Agricultural University, Beijing 100193, China; wangbo123@cau.edu.cn; 3 Animal-Centered Environments Group, Department of Biosystems and Engineering, Swedish University of Agricultural Sciences (SLU), P.O. Box 190, 234 22 Lomma, Sweden; dengsheng.sun@slu.se

**Keywords:** lambs, *Artemisia argyi* polysaccharides, cold exposure, intestinal barrier, intestinal antioxidant

## Abstract

Cold exposure severely impairs intestinal homeostasis and growth in young lambs. This study investigated the protective effects of *Artemisia argyi* polysaccharides (AAPs) on intestinal health in cold-exposed suckling lambs. Twenty-four 7-day-old lambs were randomly allocated to receive a milk replacer supplemented with 0 (Control, C), 0.5 (Low-dose, LA), 1.0 (Medium-dose, MA), or 2.0 g/kg (High-dose, HA) of AAPs for 14 days under cold conditions (−8 °C to −5 °C). The results showed that the MA group significantly increased ADG and decreased the F/G (*p* < 0.05) compared to the control. Both LA and MA groups significantly improved EE and Ca digestibility, while the MA group further increased CP and P digestibility (*p* < 0.05). The LA group enhanced jejunal chymotrypsin and lipase activities, whereas the MA group elevated chymotrypsin, lipase, and α-amylase activities (*p* < 0.05). Additionally, both LA and MA upregulated *Nrf2* mRNA expression (and downregulated *Keap1* mRNA expression in the jejunum), suggesting activation of the *Nrf2/Keap1* pathway; the MA group significantly upregulated *CAT*, *GSH-Px*, and *SOD1* mRNA expressions, enhanced SOD and CAT activities, and reduced colonic MDA content (*p* < 0.05). The MA group also significantly elevated the VH/CD across the small intestine, upregulated tight junction genes (*Claudin-1* and *Claudin-4* in jejunum; *ZO-1* and *Claudin-1* in colon), and decreased serum D-LA and DAO levels (*p* < 0.05). Cecal microbiota sequencing showed that AAPs enriched the relative abundance of *Anaerofilum* and reduced *Akkermansia*. In conclusion, 1.0 g/kg AAP supplementation effectively mitigates intestinal oxidative stress, improves barrier integrity, and modulates gut microbiota, thereby enhancing nutrient digestibility and growth performance in cold-stressed suckling lambs.

## Simple Summary

Newborn lambs raised during harsh winters often suffer from cold stress, which damages their intestines and hinders their early growth. This problem is especially severe for twin or orphaned lambs fed milk replacers, which lack the full protective benefits of mother’s milk. This study investigated whether adding a natural plant extract, *Artemisia argyi* polysaccharides (AAPs), to milk replacers could protect the gut health of cold-stressed suckling lambs. We found that supplementing the diet with a moderate dose of AAPs significantly improved the lambs’ ability to digest and absorb nutrients, leading to better daily weight gain. The extract worked effectively by boosting the body’s natural antioxidants, thereby protecting the delicate intestinal lining from cold-induced damage. Additionally, AAPs strengthened the gut barrier to prevent harmful substances from leaking into the bloodstream and promoted a healthier balance of gut bacteria. These findings demonstrate that AAPs are a highly promising, natural feed additive that can safeguard intestinal health and promote the efficient growth of young livestock facing severe cold environments.

## 1. Introduction

Endowed with abundant natural pasture resources, Inner Mongolia plays a dominant role in domestic sheep breeding and stable mutton supply, notably accounting for 26.92% of the national sheep inventory, representing half of the country’s sheep resources [1]. Intensive winter lambing and the introduction of highly prolific breeds including Hu sheep and Small-tail Han sheep are the main strategies to maximize production efficiency in the large-scale sheep industry in Inner Mongolia [2]. High rates of twinning and multiple births inevitably lead to maternal milk shortages, making milk replacers an indispensable substitute for suckling lambs [3]. While essential, feeding milk replacers presents a severe challenge to early development [4,5]. At this pre-ruminant stage of suckling lambs, the rumen is underdeveloped, rendering the intestine the primary organ for nutrient digestion and absorption. Thus, the integrity of the intestinal mucosal barrier directly determines growth potential [6]. Regrettably, compared to maternal milk, milk replacers are associated with a relatively lower intestinal absorptive capacity and mucosal barrier function [7]. This nutritional vulnerability is severely exacerbated during winter lambing, as cold exposure inevitably induces systemic oxidative stress, disrupts gut microbiota homeostasis, and damages the delicate intestinal mucosal barrier in suckling lambs [8,9]. These compounding factors of cold stress and milk replacer feeding collectively hinder nutrient utilization and suppress early growth. Therefore, it is of critical importance to identify effective nutritional interventions to mitigate intestinal damage in milk replacer-fed suckling lambs in cold weather.

Natural plant extracts, owing to their excellent biocompatibility, minimal side effects, and multi-target regulatory properties, have emerged as ideal candidates for young animals [10,11]. Extracts from *Artemisia* species (e.g., *Artemisia argyi*, *Artemisia annua*, and *Artemisia ordosica*) have antioxidant, anti-inflammatory, and intestinal-protective capacities [12,13,14,15]. *Artemisia argyi* is an indigenous plant widely distributed across northern China. *Artemisia argyi* polysaccharides (AAPs) are heteropolysaccharides rich in diverse monosaccharides, including mannose, rhamnose, glucuronic acid, galacturonic acid, and arabinose [16], which possess synergistic properties that directly counteract the precise intestinal damage previously described: mannose inhibits pyroptosis and alleviates barrier disruption [17,18]. Galacturonic acid acts as a potent antioxidant [19]. Arabinose can upregulate the expression of tight junction proteins and improve intestinal morphology [20]. It has been confirmed that AAPs improve intestinal function by modulating gut microbiota and strengthening the mucosal barrier [21]. Based on the synergistic antioxidant and mucosal-protective properties of its monosaccharides, we hypothesized that appropriate AAP supplementation in milk replacers mitigates cold stress-induced intestinal damage in suckling lambs by activating the Nrf2/Keap1 signaling pathway, thereby preserving intestinal barrier integrity and modulating gut microbiota. To test this explicit hypothesis, we systematically measured growth performance, nutrient digestibility, intestinal permeability, morphological changes, antioxidant-related gene expression, and cecal microbiota. This study provides a theoretical basis for utilizing AAPs as a functional additive to safeguard gut homeostasis in cold-stressed neonatal ruminants.

## 2. Materials and Methods

### 2.1. Artemisia argyi Polysaccharides (AAPs)

*Artemisia argyi* polysaccharides (AAPs) were extracted from *Artemisia argyi* plants harvested in Horinger County, Inner Mongolia Autonomous Region, China. The aerial parts of the plants were collected prior to flowering and dried in the shade indoors. The dried plants were cut into small pieces, pulverized using a grinder, and passed through a 40-mesh sieve to obtain a fine powder for further use. *Artemisia argyi* powder was placed in a beaker, followed by the addition of cellulase (derived from Trichoderma viride; Shanghai Yuanye Bio-Technology Co., Ltd., Shanghai, China) and distilled water. The primary purpose of the cellulase was to enzymatically degrade the plant cell wall structure, thereby facilitating the release of intracellular active polysaccharides and significantly improving the extraction yield. The mixture was incubated with constant shaking and then heated in a 70 °C water bath for 1 h to inactivate the enzyme. Subsequently, the mixture was filtered through filter paper to remove solid plant residues, and the resulting filtrate was concentrated using a rotary evaporator (RE-5298, Shanghai Yarong Biochemical Instrument Factory, Shanghai, China) under reduced pressure. Absolute ethanol (Sinopharm Chemical Reagent Co., Ltd., Shanghai, China) was then added to the concentrated extract at a volume ratio of 1:4. The mixture was thoroughly stirred and left to precipitate for 24 h. The resulting precipitate was separated and freeze-dried using a lyophilizer (ALPHA 1-2LD plus, Martin Christ, Osterode am Harz, Germany) to obtain the AAP powder, which was stored at 4 °C. Based on this optimized extraction process, the extraction yield of the AAP powder was 12.46% (i.e., yielding 124.6 g of AAP powder per kg of dry *Artemisia argyi* matter) [22].

### 2.2. Animals, Experimental Design, and Sample Collection

The animal feeding trial was conducted during the winter season (from December to February), with the average ambient temperature inside the barn ranging from −8 °C to −5 °C. All lambs were housed in individual pens. A total of 24 healthy, 7-day-old lambs with similar initial body weights were randomly allocated into four groups (six replicates per group, one lamb per replicate), with each group containing 3 males and 3 females. The lambs were fed a basal diet (Table 1) supplemented with AAP at 0 (Control group, C), 0.5 (Low-dose group, LA), 1.0 (Medium-dose group, MA), and 2.0 g/kg (High-dose group, HA) for a 14-day experimental period. The lambs were fed four times daily (08:00, 12:00, 16:00, and 20:00). At each feeding, 25 g of milk replacer powder was reconstituted, and the predetermined dosage of AAPs was thoroughly mixed in. The milk replacer was consistently consumed completely by all lambs at each feeding, and thus, no refusals were recorded. Starter feed and hay were provided ad libitum.

During the 14-day feeding trial, fasted lambs were weighed before the morning feeding on days 1, 8, and 15 to evaluate growth performance. For nutrient digestibility analysis, feces were continuously collected for 7 days (days 8 to 14). On day 8 (for assessing intestinal permeability on day 7) and on day 15 (for assessing intestinal permeability on day 14), blood samples were collected from the jugular vein prior to the morning feeding. The day 15 collection was performed following a 12 h fast and 2 h water deprivation, prior to euthanasia. Serum was separated and stored at −20 °C for subsequent analysis of intestinal permeability markers. After the lambs were anesthetized by intravenous injection of sodium pentobarbital (Sigma-Aldrich, St. Louis, MO, USA), the abdominal cavity was opened to systematically harvest intestinal samples. For histomorphological analysis, segments of the duodenum, jejunum, and ileum were collected and promptly fixed in 4% paraformaldehyde. Simultaneously, the middle segments of the jejunum and colon were rapidly snap-frozen in liquid nitrogen and stored at −80 °C for the subsequent determination of digestive enzyme activities, antioxidant status, and gene expression. Cecal contents were similarly snap-frozen and preserved for microbial analysis.

### 2.3. Growth Performance and Apparent Nutrient Digestibility

Average daily gain (ADG), average daily feed intake (ADFI), and feed-to-gain ratio (F/G) were calculated to evaluate feed utilization efficiency. The ADFI was calculated as the sum of the daily intake of milk replacer powder, starter feed, and alfalfa hay for each lamb. For milk replacer, the daily intake was equivalent to the amount offered (100 g/day), as no refusals were observed. For starter feed and hay, actual intake was calculated by subtracting refusals from the amount offered.

The apparent total tract digestibility of dietary nutrients was determined using the total fecal collection method. Feed and fecal samples were collected, weighed, and mixed systematically over 7 consecutive days (days 8 to 14). Feed and fecal samples were first oven-dried at 65 °C for 72 h until constant weight, then ground to pass through a 1 mm screen. Dry matter (DM) content was subsequently determined via oven drying (GB/T 6435-2014) [23] at 105 °C for 8 h using a DGX-9053B drying oven (Fuma Experimental Equipment Co., Ltd., Shanghai, China). Crude protein (CP) was quantified via the Kjeldahl method (GB/T 6432-2018) [24] using a K9840 auto-analyzer (Haineng Scientific Instrument Co., Ltd., Dezhou, China). Ether extract (EE) was determined via Soxhlet extraction (GB/T 6433-2006) [25] using a SOX406 analyzer (Haineng Scientific Instrument Co., Ltd., Dezhou, China). Neutral detergent fiber (NDF) and acid detergent fiber (ADF) were analyzed using the filter bag method (GB/T 20806-2006 [26] and NY/T 1459-2007 [27]) with an ANKOM A200i Fiber Analyzer (ANKOM Technology, New York, NY, USA). Calcium (Ca) was determined via the potassium permanganate method (GB/T 6436-2018) [28], and phosphorus (P) was analyzed via the spectrophotometric method (GB/T 6437-2018) [29] using a UV-visible spectrophotometer (Model: 752S, Lengguang Tech., Shanghai, China) [23,24,25,26,27,28,29,30,31].

### 2.4. Intestinal Digestive Enzyme Activity

The frozen jejunal tissues were homogenized, and the activities of chymotrypsin, trypsin, lipase, and amylase were determined according to the manufacturer’s instructions using commercial assay kits (Nanjing Jiancheng Bioengineering Institute, Nanjing, China).

### 2.5. Intestinal Permeability

Serum was separated from the collected blood samples via centrifugation. The contents of D-lactic acid (D-LA) and lipopolysaccharide (LPS), alongside the activity of diamine oxidase (DAO) in the serum, were determined using commercial kits (Genme Biological Technology Co., Ltd., Wuhan, China).

### 2.6. Histomorphology Analysis

Duodenal, jejunal and ileal samples fixed in 4% paraformaldehyde were dehydrated, sectioned using a paraffin microtome, and stained with hematoxylin and eosin (HE). After staining, the sections were air-dried, mounted with neutral balsam and coverslips, and air-dried at room temperature. Villus height (VH) was measured under 4× magnification, and crypt depth (CD) was measured under 10× magnification using image processing software associated with a Nikon E200MV biological microscope (Nikon, Tokyo, Japan).

### 2.7. mRNA Analysis

Total RNA was extracted from jejunal and colonic tissues using TRIzol reagent (TaKaRa Biotechnology Co., Ltd., Dalian, China) and subjected to reverse transcription. Total RNA was reverse-transcribed into cDNA using the Evo M-MLV Reverse Transcription Premix Kit with gDNA Eraser under the following conditions: 37 °C for 15 min, followed by 85 °C for 5 s (Hunan Accurate Bioengineering Co., Ltd., Changsha, China). Real-time PCR was performed on a Roche Light Cycler^®^ 96 Real-Time PCR System using the SYBR Green Pro TaqHS Premix qPCR Kit (Roche Holding AG, Basel, Switzerland). Each sample was analyzed in a 10 μL reaction mixture containing 5 μL of 2× SYBR Green PCR Master Mix, 0.2 μL of forward primer (0.2 μM), 0.2 μL of reverse primer (0.2 μM), 1 μL of cDNA template, and 3.6 μL of nuclease-free water. The amplification program was as follows: pre-denaturation at 95 °C for 30 s; 45 cycles of denaturation at 95 °C for 5 s, annealing at 60 °C for 30 s, and extension at 72 °C for 20 s; and a melting curve program consisting of incubation at 95 °C for 15 s, 60 °C for 1 min, and 95 °C for 15 s. All samples were run in duplicate, and melting curve analysis was performed to verify the specificity of PCR products. The expression levels of target genes were normalized to β-actin. The relative fold changes of target genes in different treatment groups compared with the control group were calculated using the 2^−ΔΔCT^ method. Primers used in this study are listed in Table 2. All primers were synthesized by Sangon Biotech Co., Ltd. (Shanghai, China).

### 2.8. Antioxidant Parameters

The activities of total antioxidant capacity (T-AOC), superoxide dismutase (SOD), catalase (CAT), and glutathione peroxidase (GSH-Px), as well as the content of malondialdehyde (MDA) in jejunal and colonic tissues, were determined according to the manufacturer’s instructions for the commercial kits (Nanjing Jiancheng Bioengineering Institute, Nanjing, China).

### 2.9. 16S rRNA Gene Amplicon Sequencing

Genomic DNA of the microbial community was extracted from cecal content samples using a DNA extraction kit provided by Omega Bio-tek (Norcross, GA, USA). The quality of the extracted DNA was verified via 1% agarose gel electrophoresis, and DNA concentration and purity were determined using a NanoDrop 2000 UV-Vis spectrophotometer (Thermo Scientific, Wilmington, NC, USA). The V3-V4 region of the 16S rRNA gene in cecal content samples was amplified using the universal primers 338F (5′-ACTCCGGGAGCAGCA-3′) and 806R (5′-GGACTACHVGGTWTCTAAT-3′). After purification, quantification and homogenization, the PCR amplicons were subjected to high-throughput sequencing on the NovaSeq 6000 platform (Illumina, San Diego, CA, USA). Singletons and chimeric sequences were removed, and the remaining sequences were clustered into operational taxonomic units (OTUs) at a 97% similarity threshold using UPARSE. Subsequently, the representative sequences of OTUs were annotated against the SSU rRNA database. The Wilcoxon rank-sum test was used to compare α-diversity indices, and Beta diversity was evaluated using QIIME software (version 1.9.1) to analyze the similarity of community structure among different samples. Linear discriminant analysis coupled with effect size (LEfSe) was applied to identify differentially abundant bacterial taxa, with an LDA effect size threshold of >3. The raw sequencing reads were submitted to the NCBI Sequence Read Archive (SRA) database under the BioProject ID PRJNA1473022.

### 2.10. Statistical Analysis

Statistical analysis was performed using SPSS software (version 26.0, IBM Corp., Armonk, NY, USA). Comparisons among the four groups were conducted via one-way analysis of variance (ANOVA) followed by Duncan’s multiple range test, and linear and quadratic regression analyses were also performed. All results are presented as the mean ± standard error of the mean (SEM). Different lowercase letters indicate significant differences (*p* < 0.05).

## 3. Results

### 3.1. Effects of Artemisia argyi Polysaccharides on the Digestibility and Growth Performance of Suckling Lambs

The four experimental groups had balanced sex distribution (3 males and 3 females per group) and comparable initial body weights (*p* > 0.05). As shown in Table 3, AAP supplementation in the diet significantly increased ADG and decreased F/G in the cold-exposed suckling lambs, demonstrating a clear quadratic response. As the inclusion level of AAPs increased, both ADG and F/G exhibited significant quadratic trends. Specifically, lambs in the MA group achieved the optimal growth response, marked by a significant increase in ADG and a concurrent reduction in F/G compared to the unsupplemented control (*p* < 0.05).

As presented in Table 4, the apparent digestibility of crude protein (CP), ether extract (EE), and calcium (Ca) followed a quadratic pattern. Relative to the control, group LA and MA significantly elevated the digestibility of EE and Ca (*p* < 0.05). Furthermore, the MA treatment maximized nutrient metabolic rates, yielding significantly higher digestibility coefficients for both CP and phosphorus (P) (*p* < 0.05).

### 3.2. Effects of Artemisia argyi Polysaccharides on the Intestinal Digestive Enzyme Activity of Suckling Lambs

**Figure 1:**
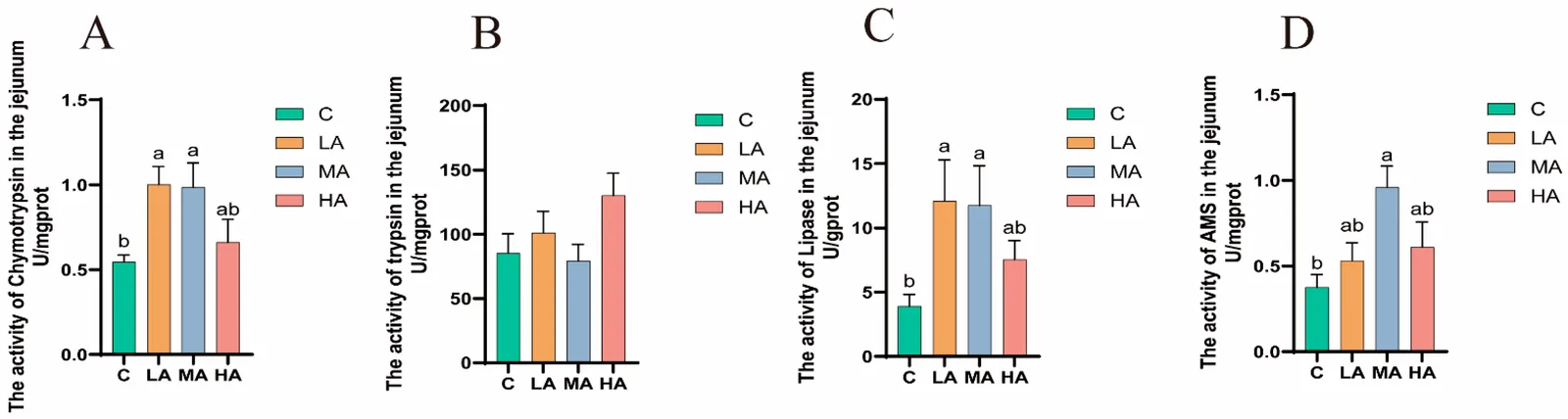
Effects of different doses of AAPs on intestinal digestive enzyme activities in the jejunum: (**A**) Jejunal chymotrypsin activity; (**B**) jejunal trypsin activity; (**C**) jejunal lipase activity; (**D**) jejunal α-amylase activity. The results are presented as Mean ± SEM. Different lowercase letters above the bars indicate significant differences (*p* < 0.05).

As shown in Figure 1, the LA diet significantly stimulated the activities of chymotrypsin and lipase compared to the control (*p* < 0.05). Notably, the MA treatment induced a broader enzymatic enhancement, resulting in significantly elevated activities of chymotrypsin, lipase, and α-amylase within the jejunal tissue (*p* < 0.05).

### 3.3. Effects of Artemisia argyi Polysaccharides on the Intestinal Permeability of Suckling Lambs

**Figure 2:**
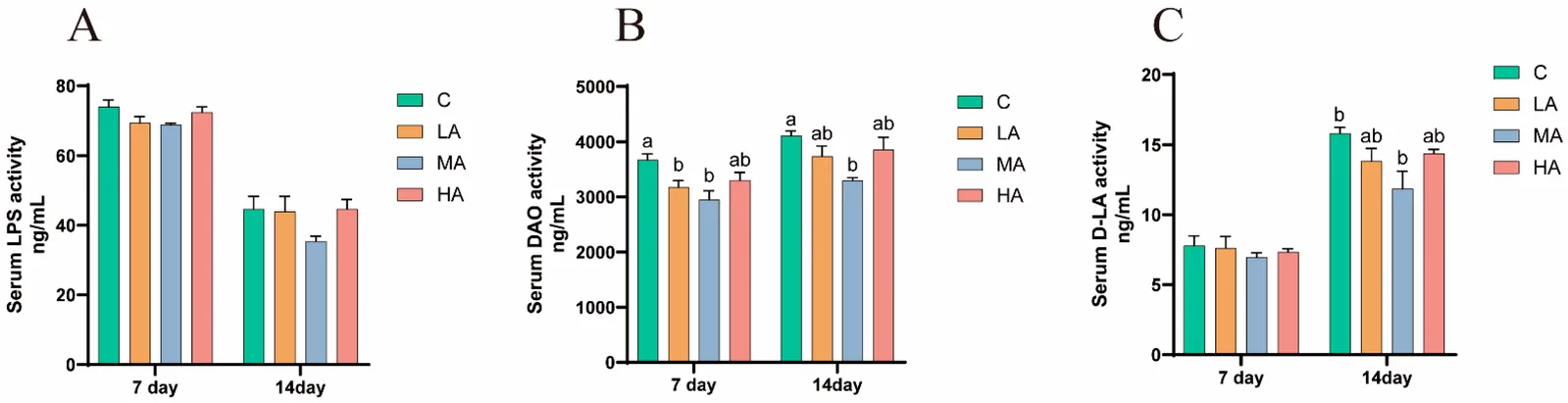
Effects of different doses of AAPs on intestinal permeability: (**A**) Serum lipopolysaccharide (LPS) content at days 7 and 14 of the trial; (**B**) serum diamine oxidase (DAO) activity at days 7 and 14 of the trial; (**C**) serum D-lactic acid (D-LA) content at days 7 and 14 of the trial. The results are presented as Mean ± SEM. Different lowercase letters above the bars indicate significant differences (*p* < 0.05).

As illustrated in Figure 2, the DAO activity in serum was significantly decreased in both the LA and MA groups compared to the control on day 7 (*p* < 0.05). Furthermore, a sustained improvement was observed on day 14, where the MA treatment resulted in significant decreases in both serum DAO activity and D-LA content (*p* < 0.05).

### 3.4. Effects of Artemisia argyi Polysaccharides on the Intestinal Villi Development of Suckling Lambs

**Figure 3:**
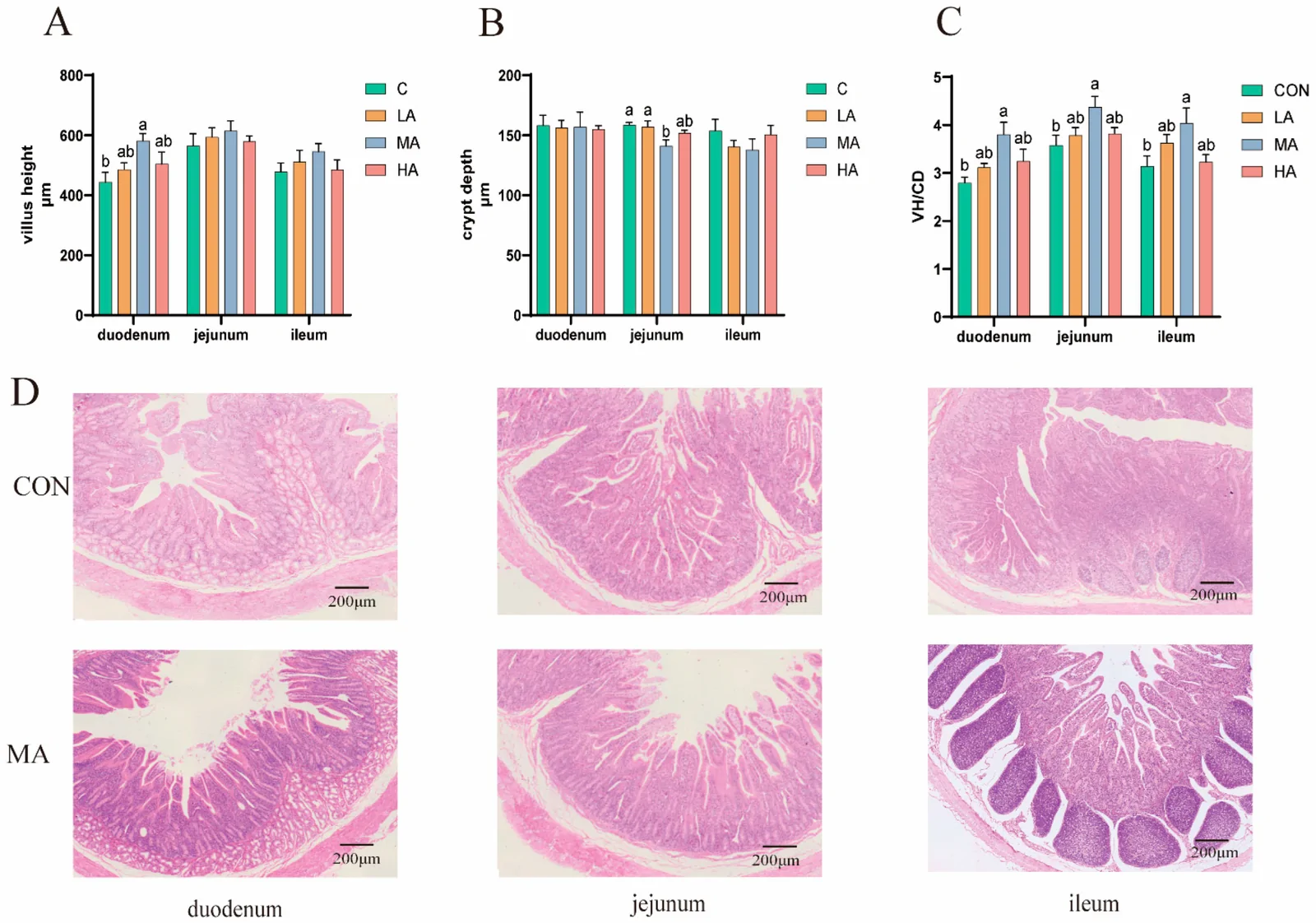
Effects of different doses of AAPs on histological changes in the duodenum, jejunum and ileum: (**A**) Intestinal villus height of lambs; (**B**) intestinal crypt depth of lambs; (**C**) intestinal VH/CD ratio of lambs; (**D**) HE staining of duodenum, jejunum, and ileum sections in group C and group MA. The results are presented as Mean ± SEM. Different lowercase letters above the bars indicate significant differences (*p* < 0.05).

As shown in Figure 3, AAP supplementation in the diet improved intestinal histomorphology, indicative of improved mucosal development and integrity. Morphometric analysis revealed that MA supplementation significantly increased duodenal VH and reduced jejunal CD relative to the control (*p* < 0.05; Figure 3A,B). Notably, although ileal morphometric parameters were not significantly altered by the treatments, AAP administration at the MA dose significantly elevated the VH/CD ratio in the duodenum, jejunum, and ileum (*p* < 0.05; Figure 3C), reflecting a comprehensive improvement in intestinal absorptive surface area.

### 3.5. Effects of Artemisia argyi Polysaccharides on the Intestinal Barrier in Suckling Lambs

**Figure 4:**
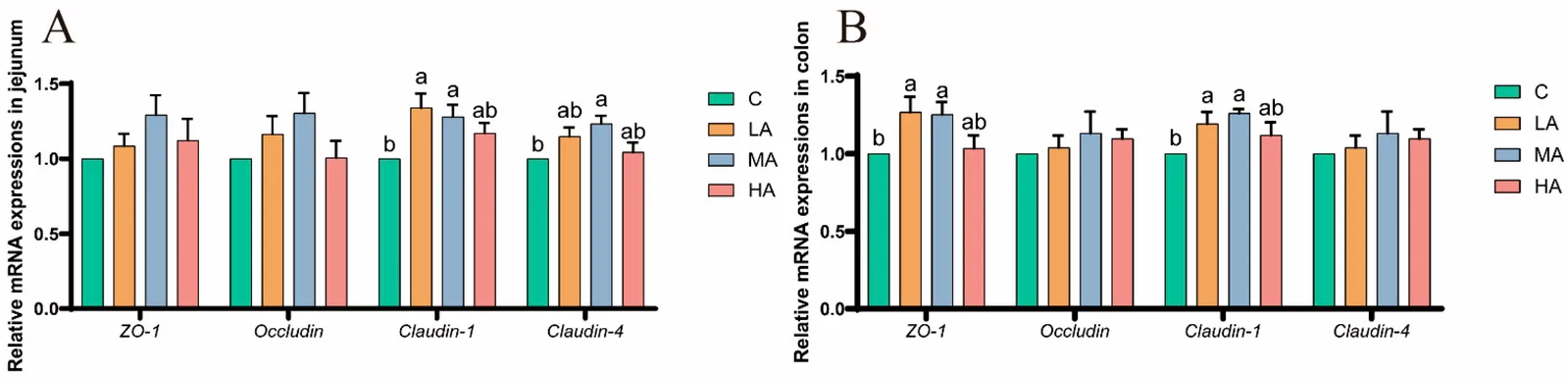
The mRNA expression of *ZO-1*, *Occludin*, *Claudin-1*, and *Claudin-4* in the jejunum and colon. (**A**,**B**) Gene expression of *ZO-1*, *Occludin*, *Claudin-1* and *Claudin-4* in jejunal and colonic tissues. The results are presented as Mean ± SEM. Different lowercase letters above the bars indicate significant differences (*p* < 0.05).

As shown in Figure 4, AAP supplementation in the diet induced a profound upregulation of *Claudin-1* and *Claudin-4*. Specifically, *Claudin-1* transcription was significantly elevated in both the LA and MA groups (*p* < 0.05) in the jejunal epithelium (Figure 4A) and AAP supplementation led to a significant upregulation of *Claudin-4* expression in suckling lambs in the MA treatment (*p* < 0.05). The LA diet significantly enhanced the mRNA levels of *ZO-1* and *Claudin-1* (*p* < 0.05) (Figure 4B). This transcriptional upregulation was most pronounced in the MA group, which exhibited a significant increase in *ZO-1* and *Claudin-1* expression (*p* < 0.05) compared to the control.

### 3.6. Effects of Artemisia argyi Polysaccharides on the Gene Expression of Nrf2/Keap1 Signaling Pathway-Related Factors in the Intestinal Tract of Suckling Lambs

**Figure 5:**
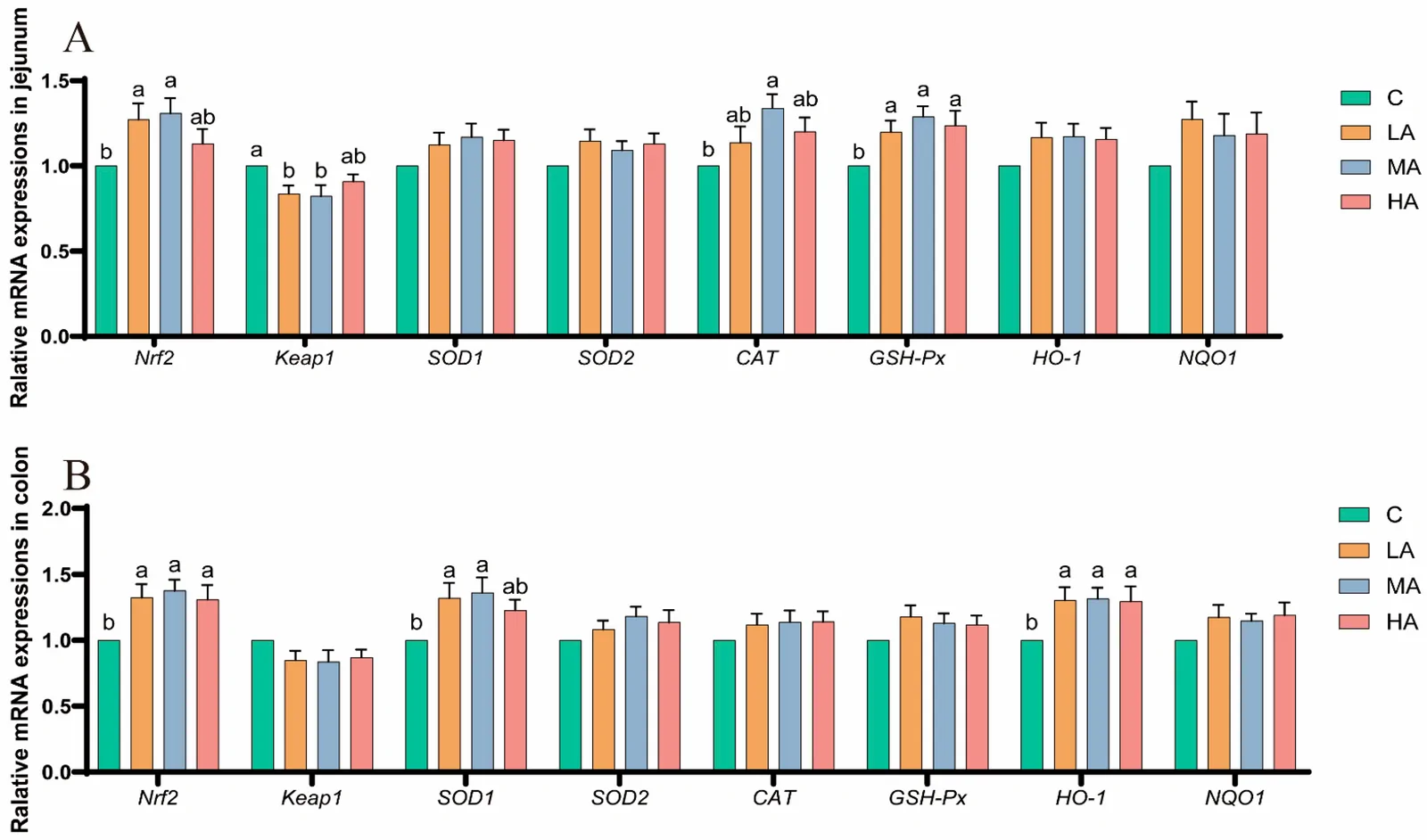
The mRNA expression of Nrf2/Keap1 signaling pathway-related factors in the jejunum and colon. (**A**,**B**) Gene expression of *Nrf2*, *Keap1*, *SOD1*, *SOD2*, *CAT*, *GSH-Px*, *HO-1*, and *NQO1* in jejunal and colonic tissues. The results are presented as Mean ± SEM. Different lowercase letters above the bars indicate significant differences (*p* < 0.05).

As shown in Figure 5, AAP supplementation in the diet activated the Nrf2/Keap1 antioxidant signaling pathway, consequently altering the expression profile of related downstream genes in the suckling lamb intestines. In the jejunum (Figure 5A), both LA and MA supplementation triggered pathway activation, evidenced by the significant upregulation of *Nrf2* and concurrent downregulation of its repressor, *Keap1* (*p* < 0.05). Consequently, downstream antioxidant gene expression was heavily promoted; notably, the MA group exhibited significant upregulations in both *CAT* and *GSH-Px* mRNA levels (*p* < 0.05). High-dose AAPs (HA), however, only yielded a significant increase in *GSH-Px* expression. In the colon (Figure 5B), AAPs similarly bolstered antioxidant defenses. The LA and MA diets significantly increased the expression of *SOD1* and *HO-1* (*p* < 0.05), with the MA treatment driving a significant upregulation of *Nrf2* (*p* < 0.05). The HA treatment also maintained elevated *Nrf2* and *HO-1* transcription (*p* < 0.05) relative to the control.

### 3.7. Effects of Artemisia argyi Polysaccharides on the Antioxidant Function of the Intestinal Tract in Suckling Lambs

**Figure 6:**
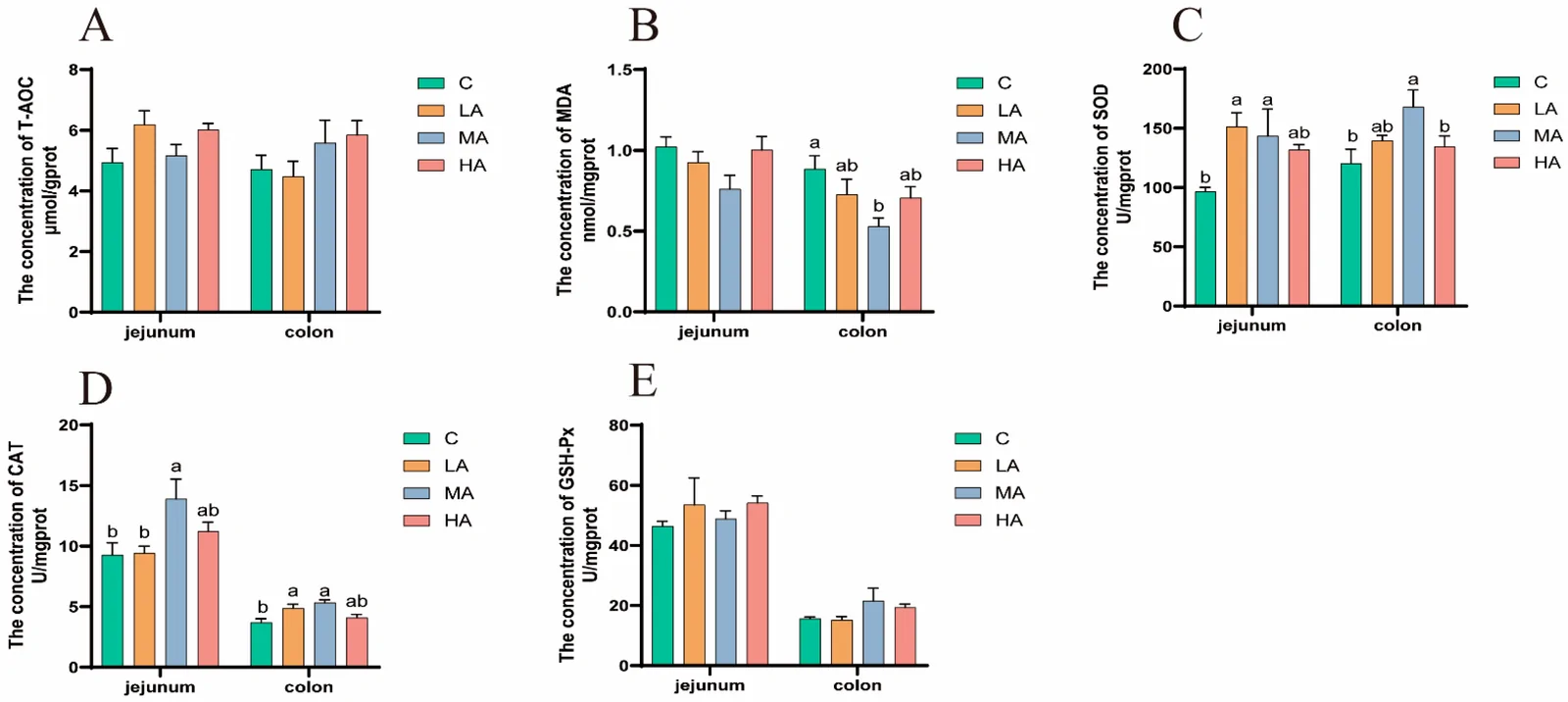
Effects of different doses of AAPs on antioxidant function in the jejunum and colon: (**A**) Total antioxidant capacity in jejunal and colonic tissues; (**B**) malondialdehyde content in jejunal and colonic tissues; (**C**) superoxide dismutase activity in jejunal and colonic tissues; (**D**) catalase activity in jejunal and colonic tissues; (**E**) glutathione peroxidase activity in jejunal and colonic tissues. The results are presented as Mean ± SEM. Different lowercase letters above the bars indicate significant differences (*p* < 0.05).

As shown in Figure 6, SOD activity was significantly elevated in the LA group (*p* < 0.05), while the MA treatment optimally enhanced the activities of both SOD and CAT compared to the control (*p* < 0.05) in jejunal tissues. Both LA and MA diets significantly increased colonic SOD and CAT activities (*p* < 0.05). The antioxidant response in the MA group translated into a significant reduction in colonic MDA content (*p* < 0.05), indicating a successful alleviation of cold-induced oxidative damage.

### 3.8. Effects of Artemisia argyi Polysaccharides on the Intestinal Microbiota of Suckling Lambs

**Figure 7:**
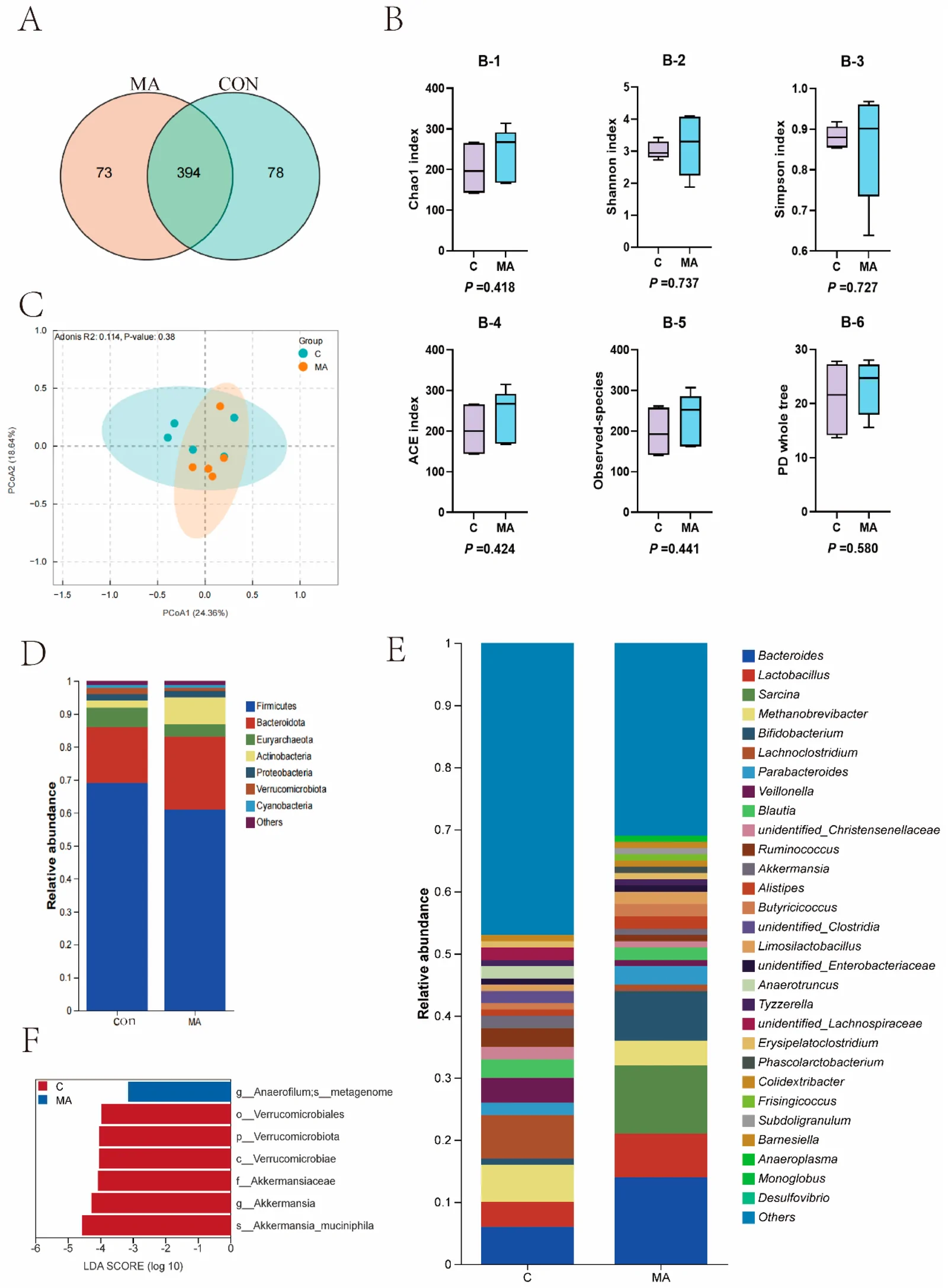
The 16S rRNA analysis of cecum microbiota between the C group and MA group. (**A**) The number of OTUs in the groups; (**B**) α diversity analysis of cecum microbiota; (**C**) PCoA analysis of cecum microbiota; (**D**) phylum-level analysis of cecum microbiota; (**E**) genus-level analysis of cecum microbiota; (**F**) LEfse analysis of cecum microbiota.

To investigate the impact of the optimal AAP dose (1.0 g/kg, MA) on gut microecology, 16S rRNA gene amplicon sequencing of cecal contents was performed. While dietary AAPs did not significantly alter microbial α-diversity indices (Figure 7B) or drive distinct separation in β-diversity via PCoA (Figure 7C), they effectively maintained the core microbial architecture, predominantly characterized by the phyla Firmicutes and Bacteroidota (Figure 7D). Despite similar overall community structures, Linear Discriminant Analysis Effect Size (LEfSe) revealed significant taxonomic shifts at the differential abundance level (LDA score > 3, *p* < 0.05; Figure 7F). Notably, the MA group was significantly enriched with specific functional taxa, including the genus *Anaerofilum* and *Phascolarctobacterium*. Conversely, taxa such as the phylum *Verrucomicrobiota* and the family *Akkermansiaceae* were significantly overrepresented in the unsupplemented control group. These results suggest that while AAPs preserve overall microbial homeostasis, they subtly remodel the relative abundance of specific cecal microbiota under cold exposure.

## 4. Discussion

Newborn lambs have an underdeveloped rumen, with the intestine serving as the major site for nutrient absorption. Intestinal barrier integrity is an essential prerequisite for maintaining growth and development in young lambs [5]. Inner Mongolia is characterized by long and severely cold winters. Cold exposure can activate the sympathetic–adrenomedullary (SAM) axis in young lambs. The SAM enhances compensatory thermogenic metabolism, disrupts mitochondrial function, induces excessive accumulation of reactive oxygen species (ROS) and oxidative stress, weakens intestinal antioxidant defense capacity, impairs the structural integrity of the intestinal mucosal barrier, and ultimately reduces nutrient digestibility and growth performance in young lambs [9,32]. Previous research in our lab suggested that AAPs possess multiple biological properties, including antioxidant activity, intestinal barrier protection, and nutrient digestion regulation, thus exhibiting great potential as functional feed additives [16,33,34]. AAPs and their monosaccharide constituents exert prominent antioxidant effects via free radical scavenging, activation of the Nrf2 signaling cascade, and upregulation of antioxidant enzyme activities [16,35]. *Artemisia*-derived polysaccharides ameliorated intestinal antioxidant function by regulating the Nrf2/Keap1 signaling pathway in animals [36,37]. In the present study, dietary supplementation of milk replacer with AAPs markedly upregulated the mRNA levels of *CAT* and *GSH-Px* in the jejunum, as well as *SOD1* and *HO-1* in the colon of cold-exposed suckling lambs. Meanwhile, AAP treatment elevated intestinal *Nrf2* expression while downregulating *Keap1* abundance in the jejunum. These results suggest that AAPs improve intestinal antioxidant capacity by activating the intestinal Nrf2/Keap1 signaling pathway. The Nrf2/Keap1 pathway represents a core regulatory cascade that controls the transcription of antioxidant-related genes. Nrf2 modulates the expression and activation of antioxidant enzymes such as SOD, CAT, and GSH-Px, thereby eliminating excessive ROS accumulation in vivo [38]. Under oxidative stress conditions, Nrf2 dissociates from Keap1 and translocates into the nucleus, where it binds to antioxidant response elements (AREs), initiates the transcription of cytoprotective factors, and upregulates the expression of downstream antioxidant genes [39,40]. Extracts from *Artemisia* plants significantly increased Nrf2 protein expression and further facilitated the transcription and expression of *HO-1* in HT22 neuronal cells [41]. In this study, low-dose (0.5 g/kg, LA) and medium-dose (1 g/kg, MA) AAP supplementation increased the activities of SOD and CAT in the jejunum and colon of cold-exposed lambs, while MA treatment reduced colonic MDA content, which is highly consistent with our previous in vitro finding that AAPs possess strong iron-reducing power and exceptional scavenging abilities against DPPH, hydroxyl, and superoxide anion radicals [22]. The observed antioxidant effects may be attributed to the structural characteristics of AAP molecules, which are known to possess free radical scavenging properties [42,43]. Numerous studies have confirmed that plant polysaccharides improved animal antioxidant status by modulating the Nrf2 signaling pathway and enhancing antioxidant enzyme activities [44,45,46]. *Artemisia ordosica* polysaccharides alleviated LPS-induced oxidative damage and improved intestinal antioxidant enzyme activities by regulating genes associated with the Nrf2/Keap1 signaling pathway [36]. Due to the esophageal groove reflex in 7-day-old pre-ruminant lambs, the liquid milk replacer along with the supplemented AAPs essentially bypassed the rumen and directly entered the abomasum, reaching the intestine to exert its biological functions. The potent antioxidant capacity of *Artemisia* polysaccharides has been associated with their structural characteristics and active functional groups [19,42,43]. Previous studies have demonstrated that *Artemisia argyi* extracts or polysaccharides enhanced antioxidant enzyme activities and the expression of antioxidant-related genes, thereby improving intestinal antioxidant capacity in broilers [47]. Additionally, *Artemisia annua* polysaccharides, which share similar monosaccharide structural characteristics with AAPs, modulated the expression of Nrf2/Keap1 pathway-related genes, significantly increased SOD and CAT activities, and effectively relieved oxidative stress induced by Escherichia coli challenge [48]. Zhao et al. [47] also reported that dietary supplementation with *Artemisia argyi* extracts improved intestinal antioxidant capacity in broilers by enhancing antioxidant enzyme activities and eliminating excess free radicals in intestinal tissues. Under the present experimental conditions, supplementation with 0.5 g/kg (LA) and 1 g/kg (MA) AAPs yielded the most favorable effects on the expression of Nrf2/Keap1 pathway-related genes and antioxidant enzyme activities in the jejunum and colon of cold-exposed lambs, with optimal antioxidant performance observed in these two groups. In summary, AAPs effectively enhanced intestinal antioxidant capacity in cold-exposed suckling lambs by modulating the transcription of Nrf2/Keap1 pathway-related genes. This modulation elevated endogenous antioxidant enzyme activities and facilitated the elimination of excessive free radicals and ROS, ultimately improving the intestinal antioxidant profile of lambs under cold stress.

The development and structural integrity of the intestinal barrier are tightly regulated by systemic oxidative stress status [49]. In cold-exposed lambs, SAM axis activation triggers blood redistribution, leading to inadequate mucosal perfusion and sustained intestinal ischemia–hypoxia. Elevated metabolism resulting from cold exposure produces abundant free radicals that disturb intestinal redox homeostasis, impair intestinal epithelial structural integrity, and cause typical morphological lesions, including villus atrophy and crypt hyperplasia. These lesions damage intestinal histological structure and barrier function, thereby reducing nutrient digestibility and absorption efficiency in lambs under cold stress [50,51,52]. *Artemisia argyi* leaf powder supplementation effectively increased intestinal villus height and villus height/crypt depth (VH/CD) ratio in animals [53]. As a potent antioxidant polysaccharide targeting the Nrf2/Keap1 cascade, AAPs, as the major active component of *Artemisia argyi*, efficiently relieved intestinal mucosal morphological damage induced by osmotic diarrhea, increased goblet cell number, and facilitated the restoration of intestinal barrier function [47]. In this study, dietary AAP supplementation increased the VH/CD ratio in the duodenum, jejunum, and ileum of cold-exposed suckling lambs in the MA group, which contributed to improved intestinal nutrient digestion and absorption, showing a dose-dependent improvement consistent with intestinal antioxidant modulation. Notably, the optimal morphological improvement of small intestinal villi was achieved at the AAP supplemental dose of 1 g/kg. The VH/CD ratio is closely correlated with the turnover dynamics of intestinal epithelial cells. Enhanced epithelial proliferation accelerates crypt cell production rate (CCPR), optimizes intestinal epithelial microstructure, and consequently elevates the VH/CD ratio [54]. *Artemisia ordosica* polysaccharides enhanced intestinal antioxidant capacity, eliminated excessive ROS, alleviated LPS-triggered oxidative damage, promoted intestinal epithelial cell proliferation, and thereby ameliorated intestinal histological morphology [36]. Additionally, fermented *Artemisia argyi* improved intestinal antioxidant function, facilitated small intestinal villus development, reduced crypt depth, and enhanced the nutrient digestibility and growth performance of animals [55]. Therefore, AAP supplementation enhanced intestinal antioxidant defense and eliminated excessive ROS in cold-exposed lambs, which efficiently alleviated oxidative stress-induced DNA damage and epithelial cell apoptosis. Meanwhile, AAPs facilitated intestinal epithelial cell proliferation and accelerated epithelial turnover, thereby increasing the small intestinal VH/CD ratio and maintaining intestinal barrier stability. These synergistic effects guaranteed efficient intestinal nutrient digestion and absorption and ultimately improved the growth performance of suckling lambs exposed to low-temperature stress.

Intestinal permeability acts as the most direct indicator of intestinal barrier integrity, as reflected by serum DAO and D-LA levels. Upregulated expression of intestinal epithelial tight junction proteins (ZO-1, Occludin and Claudins) decreases intestinal permeability [56,57], which confines DAO and D-LA to the intestinal lumen and prevents their leakage into the peripheral circulation, thereby maintaining intact intestinal barrier function [58,59,60]. However, long-term cold exposure in lambs activates the hypothalamic–pituitary–adrenal (HPA) axis and induces excessive glucocorticoid secretion with increased cortisol content. This response further suppresses the Nrf2 antioxidant signaling pathway, weakens intestinal antioxidant capacity, and promotes excessive accumulation of reactive oxygen species (ROS). Overproduced ROS initiates oxidative stress, downregulates the expression of tight junction proteins including ZO-1 and Occludin, increases intestinal permeability, and ultimately compromises intestinal mucosal barrier function [9,50]. Numerous studies have demonstrated that dietary polysaccharide supplementation improves intestinal barrier function via upregulating tight junction protein expression and decreasing intestinal permeability [61]. Existing studies have shown that *Artemisia annua* polysaccharides can enhance intestinal antioxidant capacity, alleviate oxidative stress injury, upregulate the expression of intestinal tight junction proteins, and reduce intestinal permeability, thereby maintaining intestinal barrier function by activating the Nrf2/Keap1 signaling pathway [48]. In the present study, dietary supplementation of lamb milk replacer with *Artemisia argyi* polysaccharides significantly upregulated the mRNA expression of Claudin-1 and Claudin-4 in the jejunum, as well as ZO-1 and Claudin-1 in the colon. Meanwhile, serum D-LA concentration and DAO activity were decreased. Consistently, our results revealed that AAPs effectively mitigated cold exposure-induced intestinal barrier impairment and maintained intestinal structural integrity and barrier homeostasis in suckling lambs. Moreover, the 0.5 g/kg and 1 g/kg supplementation groups showed the optimal protective effects on intestinal barrier function. In summary, cold exposure triggers HPA axis activation, Nrf2 pathway inhibition, and subsequent oxidative stress in lambs. Appropriate AAP supplementation in milk replacer efficiently alleviates cold stress-induced intestinal oxidative damage by upregulating tight junction protein expression and lowering intestinal permeability in suckling lambs. These beneficial effects are primarily attributable to the enhanced intestinal antioxidant capacity and the subsequent preservation of barrier integrity mediated by AAP supplementation.

The microbial barrier serves as a core constituent of the intestinal barrier, maintaining intestinal microecological homeostasis and blocking pathogenic bacterial invasion to protect the intestinal mucosa [62]. The rumen of neonatal lambs is immature, and the cecum acts as the primary site for hindgut fermentation and microbial colonization. The structure and diversity of bacterial communities in cecal contents directly reflect the developmental status and homeostatic level of the intestinal microbial barrier [63]. Cold exposure disturbs hindgut microbiota, decreases microbial diversity, and impairs hindgut metabolic function and intestinal homeostasis in young ruminants [32,64]. Such adverse effects are attributed to cold stress: it not only activates the neuroendocrine stress axis, downregulates tight junction protein expression, and damages the intestinal physical barrier but also reshapes the hindgut microenvironment of lambs. Cold stress suppresses the colonization of beneficial microbes such as short-chain fatty acid-producing bacteria and triggers abnormal proliferation of opportunistic pathogens, thereby disrupting bacterial community structure, decreasing alpha diversity, and eventually breaking intestinal microecological balance [32,50]. AAPs have been proven to modulate intestinal flora, facilitate the proliferation of beneficial bacteria, inhibit the growth of harmful bacteria, and optimize intestinal barrier development [65,66]. In this study, dietary AAP supplementation markedly enhanced intestinal antioxidant capacity and preserved intestinal barrier integrity in cold-exposed suckling lambs. Among all treatments, the MA group presented the best performance in intestinal antioxidant capacity, histological morphology, tight junction protein expression, and intestinal permeability. Accordingly, differences in cecal microbial diversity and richness were further compared between the control group and the 1 g/kg AAP supplementation group. LEfSe analysis revealed that 1 g/kg dietary AAP supplementation reduced the relative abundance of *Akkermansia* (Verrucomicrobia) in the cecum of cold-exposed lambs. As a pivotal symbiont colonizing the cecal mucosa, *Akkermansia* secretes glycosidase to degrade mucin for carbon and nitrogen acquisition and regulates mucin secretion as well as mucus barrier stability [67,68]. Nevertheless, excessive enrichment of *Akkermansia* induces over-degradation of the intestinal mucus layer and compromises intestinal mucosal barrier integrity [69,70]. Consistent with the present results, previous research demonstrated that plant polysaccharide intervention decreased *Akkermansia* abundance and restrained excessive mucus consumption by intestinal microorganisms [71]. Additionally, *Anaerofilum* was significantly enriched in the MA group. Belonging to Lachnospiraceae, *Anaerofilum* is a crucial butyrate-producing beneficial bacterium in the ruminant hindgut. It optimizes energy supply for intestinal epithelial cells, strengthens intestinal barrier function, and stabilizes hindgut microecology [72,73]. Supplementation with *Artemisia ordosica* polysaccharides elevated the relative abundance of ruminal Lachnospiraceae in cashmere goats; this family was positively correlated with antioxidant indicators and negatively correlated with feed conversion ratio. Increased Lachnospiraceae abundance improved antioxidant status, maintained intestinal barrier integrity, and promoted feed utilization efficiency [15]. Taken together, these results confirm the regulatory role of AAPs in modulating intestinal development under cold exposure. Under the current experimental conditions, AAP supplementation upregulated the abundance of beneficial taxa represented by *Anaerofilum* and downregulated *Akkermansia*-related microbes. AAPs remodel intestinal flora composition by alleviating cold stress-induced gut microbiota dysbiosis in lambs, thereby preserving the intestinal microbial barrier, stabilizing intestinal homeostasis, and sustaining intestinal health.

Intestinal redox homeostasis and the structural integrity of the mucosal barrier are indispensable for maintaining normal digestive function in the small intestine [74]. Systemic stress induced by cold exposure compromises intestinal barrier integrity and disturbs intestinal antioxidant equilibrium, concomitant with retarded growth and suppressed nutrient digestion and absorption in lambs [9,50,75]. Cold exposure leads to an increase in the animal’s basal metabolic rate, with a large amount of nutrients and energy being prioritized for maintaining thermoregulation. This results in an insufficient supply of substances for growth, development, and nutrient deposition, thereby hindering the growth and development of lambs [9]. Furthermore, endocrine disturbances triggered by cold stress inhibit the synthesis and secretion of gastrointestinal digestive enzymes, preventing the adequate digestion and absorption of nutrients such as proteins, carbohydrates, and fats in the diet and reducing the apparent digestibility of these nutrients [75]. Accumulating evidence has validated that solid-state fermented *Artemisia argyi* residue (AARF) mitigates *Clostridium perfringens*-triggered oxidative damage, decreases intestinal permeability, elevates jejunal digestive enzyme activities, facilitates nutrient digestion and absorption, and ultimately enhances growth performance in broilers via activating the Nrf2 antioxidant signaling pathway [76]. Dietary supplementation with enzymatically hydrolyzed *Artemisia annua* also efficiently attenuates heat stress-induced intestinal oxidative impairment and upregulates jejunal digestive enzyme activities [77]. Dietary supplementation with AAP markedly increased the activities of jejunal chymotrypsin and lipase in the LA and MA groups and upregulated jejunal α-amylase activity in the MA group. Meanwhile, AAP supplementation improved the apparent digestibility of fat and calcium in the LA and MA groups, as well as protein and phosphorus digestibility in the MA group. Additionally, AAPs increased ADG and decreased F/G in the MA group. Overall, AAPs efficiently enhanced intestinal digestive enzyme activities and nutrient digestibility, thereby improving growth performance and feed utilization efficiency in cold-exposed suckling lambs. These outcomes are principally ascribed to AAP-mediated improvement in intestinal antioxidant capacity and barrier function, which mitigates oxidative stress-induced injury to intestinal secretory cells and maintains the normal synthesis, secretion, and biological function of digestive enzymes. The jejunum is recognized as the key intestinal segment responsible for polysaccharide-modulated regulation of digestive enzyme activities, and its enzymatic activity profile can reliably reflect intestinal protein degradation capacity and nutrient metabolic status [78]. Polysaccharides derived from *Artemisia ordosica* exert beneficial effects on improving nutrient digestibility in goats [15]. The potential mechanism is that *Artemisia* plant extracts hydrolyze macronutrients into absorbable small-molecule compounds by modulating intestinal digestive enzymes, thereby maximizing dietary nutrient utilization and optimizing animal growth performance [79]. *Artemisia annua* aqueous extract enhances the apparent digestibility of crude protein, crude fat, and dry matter by upregulating small intestinal digestive enzyme activities and concurrently lowers ADFI and F/G in sheep [80]. Similarly, dietary supplementation with *Artemisia absinthium* L. also enhances nutrient digestibility, promotes nutrient digestion and absorption, and ameliorates growth performance in sheep [81]. In summary, AAPs supplementation strengthens intestinal antioxidant capacity and maintains intestinal barrier integrity, which sustains a stable mucosal structure and intestinal microenvironment for cold-exposed lambs and further guarantees the normal secretion of digestive enzymes and transmembrane nutrient transport. A positive correlation between nutrient apparent digestibility and digestive enzyme activities was identified in the present work. By upregulating the activities of chymotrypsin, lipase, and α-amylase, AAPs facilitate the degradation and absorption of multiple nutrients through the synergistic effects of digestive enzymes, thereby comprehensively improving nutrient digestibility and utilization efficiency, optimizing growth performance, and lowering F/G in lambs. This work provides a theoretical reference for the modulation of intestinal barrier function and nutrient metabolism, as well as the healthy rearing of suckling lambs suffering cold stress.

## 5. Conclusions

In summary, dietary supplementation with 1.0 g/kg *Artemisia argyi* polysaccharides (AAPs) serves as an effective nutritional strategy to mitigate cold-induced growth retardation and intestinal impairment in suckling lambs. Mechanistically, AAPs bolster intestinal antioxidant defenses via the activation of the Nrf2/Keap1 signaling pathway. Antioxidant response consequently preserves mucosal morphology, upregulates tight junction expression, and reduces permeability, thereby reinforcing overall barrier integrity. AAPs optimize cecal microbial composition and stimulate jejunal digestive enzyme activities, which synergistically maximize nutrient digestibility. Collectively, these findings highlight the strong potential of AAPs as a natural, functional feed additive to safeguard gut homeostasis and promote the efficient rearing of neonatal ruminants under challenging cold stress conditions.

## Figures and Tables

**Table 1:** The composition and level of basal diet (dry matter basis).

Items	Starter Pellets	Milk Replacer	Alfalfa Hay
Corn	40.00	-	-
DDGS	3.00	-	-
Soybean meal	26.00	-	-
Wheat bran	13.00	-	-
Corn germ meal	6.00	-	-
Soybean hulls	4.00	-	-
Extruded soybean	4.00	-	-
Limestone	2.00	-	-
CaHPO_4_	0.50	-	-
NaCl	0.50		-
Premix ^1^	1.00	-	-
Total	100.00	-	-
Nutrient level			
DM (%)	89.86	93.23	86.76
CP (%)	21.78	19.44	14.81
EE (%)	4.68	11.54	2.42
NDF (%)	22.23	-	55.98
ADF (%)	8.05	-	43.04
Ash (%)	7.71	3.03	6.77
Ca (%)	1.24	1.03	1.22
P (%)	0.61	0.59	0.18
ME (MJ/kg)	13.68	16.94	9.67

^1^ The premix provided the following per kg of diets: VA, 800,000 IU, VD 30,000 IU, VE 3000 mg; Cu 0.8 g, Fe 4 g, Mn 4 g, Zn 5 g, I 70 mg, Se 20 mg, and Co 40 mg; DM, dry matter; CP, crude protein; EE, Ether Extract; NDF, neutral detergent fiber; ADF, acid detergent fiber; Ca, calcium; P, phosphorus; ME, metabolizable energy. All nutrient values are expressed as percentages (%), except for ME, which is expressed as MJ/kg.

**Table 2:** Primers used in quantitative real-time PCR analysis.

Gene	Length, bp	NCBI Accession.	Nucleotide Sequence of Primers (5′→3′)
*β-actin*	208	NM_001009784.2	F: GTCACCAACTGGGACGCAA
			R: AGGCGTACAGGGACAGCA
*ZO-1*	161	XM_018066114.1	F: CGACCAGATCCTCAGGGTAA
			R: AATCACCCACATCGGATTCT
*Occludin*	212	XM_018065677.1	F: CCATACCACTCCTCCTCCGTA
			R: GTGGACTTTCAAAAGGCCTGG
*Claudin-1*	161	XM_005675123.3	F: CTGCCCCAGTGGAAGGTTTA
			R: GTTGCTTGCAGAGTGCTGTTC
*Claudin-4*	237	XM_005697785.2	F: AAGGTGTACGACTCGCTGCT
			R: GACGTTGTTAGCCGTCCAG
*Nrf2*	88	XM_004004557.1	F: TGTGGAGGAGTTCAACGAGC
			R: CGCCGCCATCTTGTTCTTG
*Keap1*	157	XM_027969637.2	F: TTCAACAGCGAAAGTCAGGC
			R: TGCGTAGCCTCCGATACTCT
*SOD1*	182	NM_001145185	F: GGAGACCTGGGCAATGTGAA
			R: CCTCCAGCGTTTCCAGTGAA
*SOD2*	116	NM_001280703.1	F: AAACCGTCAHCCTTACACC
			R: ACAAGCCACGCTCAGAAAC
*CAT*	148	XM_004016396	F: GAGCCCACCTGCAAAGTTCT
			R: CTCCTACTGGATTACCGGCG
*GSH-Px*	163	XM_004018462.1	F: TGGTCGTACTCGGCTTCCC
			R: AGCGGATGCGCCTTCTCG
*HO-1*	74	XM_027967703.2	F: CGATGGGTCCTCACACTCAG
			R: CACACTCGCATTCACATGGC
*NQO1*	296	XM_004015102.5	F: CTCTGGCCAATTCAGAGTGG
			R: TCCATTGGGATGGACTTGCC

**Table 3:** Effects of AAP addition on growth performance of lambs.

Items ^1^	C	LA	MA	HA	SEM ^2^	Linear	Quadratic
IBW (kg)	4.05	4.27	4.03	3.95	0.15	0.695	0.823
FBW (kg)	4.46	4.80	4.67	4.41	0.15	0.833	0.593
ADG (g)	29.17 ^b^	38.10 ^ab^	45.24 ^a^	32.74 ^ab^	2.19	0.373	0.025
ADFI (g)	131.43	130.20	135.50	134.55	2.51	0.525	0.821
F/G	4.59 ^a^	3.62 ^ab^	3.20 ^b^	4.25 ^ab^	0.19	0.429	0.019

^1^ IBW, Initial Body Weight; FBW, Final Body Weight; ADG, Average Daily Gain; ADFI, Average Daily Feed Intake (including milk replacer intake, starter intake, and hay intake); F/G, Feed conversion ratio. C, control group; C, basal diet + 0 g/kg AAP; LA, basal diet + 0.5 g/kg AAP; MA, basal diet + 1 g/kg AAP; HA, basal diet + 2 g/kg AAP. ^2^ SEM, standard error of mean. ^a, b^ Values in the same row with different letter superscripts indicate significant differences (*p* < 0.05). Results are presented as Mean ± SEM.

**Table 4:** Apparent total tract digestibility of nutrients in cold-exposed lambs.

Items ^1^	C	LA	MA	HA	SEM	Linear	Quadratic
DM (%)	69.67	72.20	72.13	71.29	0.78	0.515	0.477
CP (%)	67.95 ^b^	70.70 ^ab^	73.16 ^a^	71.05 ^ab^	0.68	0.051	0.023
EE (%)	67.30 ^b^	71.87 ^a^	73.34 ^a^	69.59 ^ab^	0.80	0.255	0.011
NDF (%)	52.03	55.82	55.73	53.56	0.73	0.502	0.091
ADF (%)	39.68	42.00	42.40	40.04	0.97	0.868	0.495
Ash (%)	59.22	61.49	60.77	59.50	0.58	0.979	0.325
Ca (%)	44.70 ^b^	49.00 ^a^	50.06 ^a^	45.65 ^ab^	0.79	0.592	0.013
P (%)	35.69 ^b^	38.80 ^ab^	40.62 ^a^	38.09 ^ab^	0.74	0.181	0.060

^1^ DM, Dry Matter; CP, Crude Protein; EE, Ether Extract; NDF, Neutral Detergent Fiber; ADF, Acid Detergent Fiber; Ca, Calcium; P, Phosphorus. C, control group (basal diet + 0 g/kg AAP); LA, basal diet + 0.5 g/kg AAP; MA, basal diet + 1 g/kg AAP; HA, basal diet + 2 g/kg AAP. SEM, standard error of mean. ^a, b^ Values in the same row with different letter superscripts indicate significant differences (*p* < 0.05). Results are presented as Mean ± SEM.

## Data Availability

The raw 16S rRNA gene amplicon sequencing data generated in this study have been deposited in the NCBI Sequence Read Archive (SRA) database under the BioProject accession number PRJNA1473022. All other data supporting the findings of this study are available from the corresponding author upon reasonable request.

## References

[1] Zhang Y., Wang G., Zhang Y., Zhao S., Han C. (2021). Climate Change Is Likely to Alter Sheep and Goat Distributions in Mainland China. Front. Environ. Sci..

[2] Xue L., Chen J., Li B., Zhou S., Guo H., Zhang L., Li J., Wang Z. (2025). Genetic Diversity Assessment of Guangling Large-Tail Sheep and Introduced Breeds in Shanxi Province Using Microsatellite Markers and Inference of Heterosis Potential. Anim. Biotechnol..

[3] Herath H.M.G.P., Pain S.J., Kenyon P.R., Blair H.T., Morel P.C.H. (2021). Growth and Body Composition of Artificially-Reared Lambs Exposed to Three Different Rearing Regimens. Animals.

[4] Yao Y., Cai X., Ye Y., Wang F., Chen F., Zheng C. (2021). The Role of Microbiota in Infant Health: From Early Life to Adulthood. Front. Immunol..

[5] Dunière L., Ruiz P., Lebbaoui Y., Guillot L., Bernard M., Forano E., Chaucheyras-Durand F. (2023). Effects of Rearing Mode on Gastro-Intestinal Microbiota and Development, Immunocompetence, Sanitary Status and Growth Performance of Lambs from Birth to Two Months of Age. Anim. Microbiome.

[6] Meale S.J., Chaucheyras-Durand F., Berends H., Guan L.L., Steele M.A. (2017). From Pre- to Postweaning: Transformation of the Young Calf’s Gastrointestinal Tract. J. Dairy Sci..

[7] Hammon H., Blum J.W. (1997). Prolonged Colostrum Feeding Enhances Xylose Absorption in Neonatal Calves. J. Anim. Sci..

[8] Price E.R., Ruff L.J., Guerra A., Karasov W.H. (2013). Cold Exposure Increases Intestinal Paracellular Permeability to Nutrients in the Mouse. J. Exp. Biol..

[9] Shi L., Xu Y., Jin X., Wang Z., Mao C., Guo S., Yan S., Shi B. (2022). Influence of Cold Environments on Growth, Antioxidant Status, Immunity and Expression of Related Genes in Lambs. Animals.

[10] Ning C., Jiao Y., Wang J., Li W., Zhou J., Lee Y.-C., Ma D.-L., Leung C.-H., Zhu R., David Wang H.-M. (2022). Recent Advances in the Managements of Type 2 Diabetes Mellitus and Natural Hypoglycemic Substances. Food Sci. Hum. Wellness.

[11] Chen X., Sun W., Xu B., Wu E., Cui Y., Hao K., Zhang G., Zhou C., Xu Y., Li J. (2021). Polysaccharides from the Roots of Millettia Speciosa Champ Modulate Gut Health and Ameliorate Cyclophosphamide-Induced Intestinal Injury and Immunosuppression. Front. Immunol..

[12] Ma Q., Tan D., Gong X., Ji H., Wang K., Lei Q., Zhao G. (2022). An Extract of *Artemisia argyi* Leaves Rich in Organic Acids and Flavonoids Promotes Growth in BALB/c Mice by Regulating Intestinal Flora. Animals.

[13] Nurlybekova A., Kudaibergen A., Kazymbetova A., Amangeldi M., Baiseitova A., Ospanov M., Aisa H.A., Ye Y., Ibrahim M.A., Jenis J. (2022). Traditional Use, Phytochemical Profiles and Pharmacological Properties of *Artemisia* Genus from Central Asia. Molecules.

[14] Gang G., Gao R., Zhao H., Xu Y., Xing Y., Jin X., Hong L., Yan S., Shi B. (2024). Effects of Water Extracts of *Artemisia annua* L. on Rumen Immune and Antioxidative Indexes, Fermentation Parameters and Microbials Diversity in Lambs. Front. Microbiol..

[15] Li S., Guo Y., Guo X., Shi B., Ma G., Yan S., Zhao Y. (2023). Effects of *Artemisia ordosica* Crude Polysaccharide on Antioxidant and Immunity Response, Nutrient Digestibility, Rumen Fermentation, and Microbiota in Cashmere Goats. Animals.

[16] Zhang P., Sun H., Yang D., Wang Y., Cheng J., Zeng C. (2023). Oral Administration of *Artemisia argyi* Polysaccharide Increases Estrogen Level and Maintains Blood Lipid Homeostasis in Ovariectomized Rats. Pharmacology.

[17] Xiao P., Hu Z., Lang J., Pan T., Mertens R.T., Zhang H., Guo K., Shen M., Cheng H., Zhang X. (2022). Mannose Metabolism Normalizes Gut Homeostasis by Blocking the TNF-α-Mediated Proinflammatory Circuit. Cell. Mol. Immunol..

[18] Ai Y., Wang W., Liu F., Fang W., Chen H., Wu L., Hong X., Zhu Y., Zhang C., Liu L. (2023). Mannose Antagonizes GSDME-Mediated Pyroptosis Through AMPK Activated by Metabolite GlcNAc-6P. Cell Res..

[19] Wang H., Yan R., Cheng H., Zou M., Wang H., Zheng K. (2023). Hollow Glass Microspheres/Phenolic Syntactic Foams with Excellent Mechanical and Thermal Insulate Performance. Front. Chem..

[20] Kang L., Pang J., Zhang X., Liu Y., Wu Y., Wang J., Han D. (2023). L-Arabinose Attenuates LPS-Induced Intestinal Inflammation and Injury Through Reduced M1 Macrophage Polarization. J. Nutr..

[21] Du H., Xing Y., Xu Y., Jin X., Yan S., Shi B. (2023). Dietary *Artemisia ordosica* Polysaccharide Enhances Spleen and Intestinal Immune Response of Broiler Chickens. Biology.

[22] Zhang X., Jiang M., Xing Z., Han Z., Li J., Jin X. (2025). Optimization of Extraction Process of *Artemisia argyi* Poly-Saccharide by Response Surface Methodology and Analysis of Its Components and In Vitro Antioxidant Activity. Chin. J. Anim. Nutr..

[23] (2014). Determination of Moisture in Feeds.

[24] (2018). Determination of Crude Protein in Feeds.

[25] (2006). Determination of Ether Extract in Feeds.

[26] (2006). Determination of Neutral Detergent Fiber in Feedstuffs.

[27] (2007). Determination of Acid Detergent Fiber in Feedstuff.

[28] (2018). Determination of Calcium in Feeds.

[29] (2018). Determination of Phosphorus in Feeds—Spectrophotometry.

[30] (2007). Determination of Crude Ash in Feeds.

[31] (2023). Code of Practice for Livestock and Poultry Slaughtering Operation—Sheep and Goat.

[32] Cheng X., Liang Y., Ji K., Feng M., Du X., Jiao D., Wu X., Zhong C., Cong H., Yang G. (2025). Enhanced Propionate and Butyrate Metabolism in Cecal Microbiota Contributes to Cold-Stress Adaptation in Sheep. Microbiome.

[33] Lan M.-B., Zhang Y.-H., Zheng Y., Yuan H.-H., Zhao H.-L., Gao F. (2010). Antioxidant and Immunomodulatory Activities of Polysaccharides from Moxa (*Artemisia argyi*) Leaf. Food Sci. Biotechnol..

[34] Ma G.-F., Zhao B.-B., Tang P., Tian D., Zhao Y., Liu Z., Chen L.-L. (2025). Structural Characterization and Immunomodulatory Activity of Polysaccharides from the Roots of *Artemisia argyi*. Carbohydr. Polym..

[35] Hu W., Yu A., Bi H., Gong Y., Wang H., Kuang H., Wang M. (2024). Recent Advances in *Artemisia argyi* Levl. et Vant. Polysaccharides: Extractions, Purifications, Structural Characteristics, Pharmacological Activities, and Existing and Potential Applications. Int. J. Biol. Macromol..

[36] Xing Y.Y., Zheng Y.K., Yang S., Zhang L.H., Guo S.W., Shi L.L., Xu Y.Q., Jin X., Yan S.M., Shi B.L. (2021). *Artemisia ordosica* Polysaccharide Alleviated Lipopolysaccharide-Induced Oxidative Stress of Broilers via Nrf2/Keap1 and TLR4/NF-κB Pathway. Ecotoxicol. Environ. Saf..

[37] Gang G., Gao R., Zhao H., Jin X., Xing Y., Hong L., Yan S., Xu Y., Shi B. (2025). Effects of Diet Supplemented with Water Extracts of *Artemisia annua* L. on Small Intestinal Immune and Antioxidative Indexes in Lambs. Front. Vet. Sci..

[38] Bellezza I., Giambanco I., Minelli A., Donato R. (2018). Nrf2-Keap1 Signaling in Oxidative and Reductive Stress. Biochim. Biophys. Acta Mol. Cell Res..

[39] Kim J., Cha Y.-N., Surh Y.-J. (2010). A Protective Role of Nuclear Factor-Erythroid 2-Related Factor-2 (Nrf2) in Inflammatory Disorders. Mutat. Res. Fundam. Mol. Mech. Mutagen..

[40] McMahon M., Lamont D.J., Beattie K.A., Hayes J.D. (2010). Keap1 Perceives Stress via Three Sensors for the Endogenous Signaling Molecules Nitric Oxide, Zinc, and Alkenals. Proc. Natl. Acad. Sci. USA.

[41] Uy N.P., He M.T., Lee C.-D., Lee Y., Kang K.S., Lee S. (2025). Artemisia Extracts and Phytochemicals Inhibit Glutamate-Induced Oxidative Cell Death and Upregulate the Nrf2/HO-1 Signaling Pathway in HT22 Neuronal Cells. J. Ethnopharmacol..

[42] Wang Q., Zhou X., Gou H., Chang H., Lan J., Li J., Li Z., Gao M., Wang Z., Yi Y. (2023). Antibacterial Activity of a Polysaccharide Isolated from *Artemisia argyi* Leaf against Staphylococcus Aureus and Mechanism Investigation. Int. J. Biol. Macromol..

[43] Kang Y., Li P., Zeng X., Chen X., Xie Y., Zeng Y., Zhang Y., Xie T. (2019). Biosynthesis, Structure and Antioxidant Activities of Xanthan Gum from *Xanthomonas campestris* with Additional Furfural. Carbohydr. Polym..

[44] Wang L., Xie Y., Yang W., Yang Z., Jiang S., Zhang C., Zhang G. (2019). Alfalfa Polysaccharide Prevents H_2_O_2_-Induced Oxidative Damage in MEFs by Activating MAPK/Nrf2 Signaling Pathways and Suppressing NF-κB Signaling Pathways. Sci. Rep..

[45] Li S., Song Z., Liu T., Liang J., Yuan J., Xu Z., Sun Z., Lai X., Xiong Q., Zhang D. (2018). Polysaccharide from *Ostrea rivularis* Attenuates Reproductive Oxidative Stress Damage via Activating Keap1-Nrf2/ARE Pathway. Carbohydr. Polym..

[46] Wang K., Yang L., Zhou J., Pan X., He Z., Liu J., Zhang Y. (2022). *Smilax china* L. Polysaccharide Alleviates Oxidative Stress and Protects from Acetaminophen-Induced Hepatotoxicity via Activating the Nrf2-ARE Pathway. Front. Pharmacol..

[47] Zhao F., Shi B., Sun D., Chen H., Tong M., Zhang P., Guo X., Yan S. (2016). Effects of Dietary Supplementation of *Artemisia argyi* Aqueous Extract on Antioxidant Indexes of Small Intestine in Broilers. Anim. Nutr..

[48] Guo S., Shi B., Xing Y., Xu Y., Jin X., Hong L., Zhang S., Qiao M., Yan S. (2024). *Artemisia annua* L. Polysaccharide Improves the Growth Performance and Intestinal Barrier Function of Broilers Challenged with *Escherichia coli*. Front. Microbiol..

[49] Li H., Ran T., He Z., Yan Q., Tang S., Tan Z. (2016). Postnatal Developmental Changes of the Small Intestinal Villus Height, Crypt Depth and Hexose Transporter mRNA Expression in Supplemental Feeding and Grazing Goats. Small Rumin. Res..

[50] Guo J., Hu H., Chen Z., Xu J., Nie J., Lu J., Ma L., Ji H., Yuan J., Xu B. (2022). Cold Exposure Induces Intestinal Barrier Damage and Endoplasmic Reticulum Stress in the Colon via the SIRT1/Nrf2 Signaling Pathway. Front. Physiol..

[51] Lei L., Jing M., Yingce Z., Pei Z., Yun L. (2023). Selenium Deficiency Causes Oxidative Stress and Activates Inflammation, Apoptosis, and Necroptosis in the Intestine of Weaned Calves. Metallomics.

[52] Alexander G., Bell A.W., Setchell B.P. (1972). Regional Distribution of Cardiac Output in Young Lambs: Effect of Cold Exposure and Treatment with Catecholamines. J. Physiol..

[53] Chen J., Chen F., Peng S., Ou Y., He B., Li Y., Lin Q. (2022). Effects of *Artemisia argyi* Powder on Egg Quality, Antioxidant Capacity, and Intestinal Development of Roman Laying Hens. Front. Physiol..

[54] Ichikawa H., Kuroiwa T., Inagaki A., Shineha R., Nishihira T., Satomi S., Sakata T. (1999). Probiotic Bacteria Stimulate Gut Epithelial Cell Proliferation in Rat. Dig. Dis. Sci..

[55] Ni Z., Nie A., Imran M., Sun Z., Zhang B., Wang Z., Qi Y., Li C. (2025). Dietary Fermented *Artemisia argyi* Enhances Growth Performance, Hepatic Antioxidant Activity, and Intestinal Health in Hongyu Roosters. Poult. Sci..

[56] Izuddin W.I., Loh T.C., Foo H.L., Samsudin A.A., Humam A.M. (2019). Postbiotic *L. plantarum* RG14 Improves Ruminal Epithelium Growth, Immune Status and Upregulates the Intestinal Barrier Function in Post-Weaning Lambs. Sci. Rep..

[57] Gu M., Fan R., Dai X., Gu C., Wang A., Wei W., Yang S. (2023). Tannic Acid Induces Intestinal Dysfunction and Intestinal Microbial Dysregulation in Brandt’s Voles (*Lasiopodomys brandtii*). Animals.

[58] Yang W., Guo G., Chen J., Wang S., Gao Z., Zhao Z., Yin F. (2022). Effects of Dietary Fucoidan Supplementation on Serum Biochemical Parameters, Small Intestinal Barrier Function, and Cecal Microbiota of Weaned Goat Kids. Animals.

[59] Ren G., Xu L., Lu T., Zhang Y., Wang Y., Yin J. (2019). Protective Effects of Lentinan on Lipopolysaccharide Induced Inflammatory Response in Intestine of Juvenile Taimen (*Hucho taimen*, Pallas). Int. J. Biol. Macromol..

[60] Macura B., Kiecka A., Szczepanik M. (2024). Intestinal Permeability Disturbances: Causes, Diseases and Therapy. Clin. Exp. Med..

[61] Xie S.-Z., Liu B., Ye H.-Y., Li Q.-M., Pan L.-H., Zha X.-Q., Liu J., Duan J., Luo J.-P. (2019). *Dendrobium huoshanense* Polysaccharide Regionally Regulates Intestinal Mucosal Barrier Function and Intestinal Microbiota in Mice. Carbohydr. Polym..

[62] Arshad M.A., Hassan F., Rehman M.S., Huws S.A., Cheng Y., Din A.U. (2021). Gut Microbiome Colonization and Development in Neonatal Ruminants: Strategies, Prospects, and Opportunities. Anim. Nutr..

[63] Bi Y., Tu Y., Zhang N., Wang S., Zhang F., Suen G., Shao D., Li S., Diao Q. (2021). Multiomics Analysis Reveals the Presence of a Microbiome in the Gut of Fetal Lambs. Gut.

[64] Luo T., Zhu J., Li K., Li Y., Li J., Chen Y., Shi H. (2024). Crosstalk between Innate Immunity and Rumen-Fecal Microbiota under the Cold Stress in Goats. Front. Immunol..

[65] Zhang P., Yang D., Xiao J., Hong W., Sun H., Xie Q., Zeng C. (2024). *Artemisia argyi* Polysaccharide Alleviates Osmotic Diarrhea by Enhancing Intestinal Barrier Protection and Anti-Inflammation. Int. J. Biol. Macromol..

[66] Song Q., Wu H., Weng S., Wang Y., Kong L., Liu Z., Zhang K. (2024). *Artemisia argyi* Polysaccharide Ameliorates Hyperglycemia Through Modulating Gut Microbiota and Serum Metabolism in Type 2 Diabetic Mice. J. Funct. Foods.

[67] Wang Y., Li C., Li J., Zhang S., Zhang Q., Duan J., Guo J. (2024). *Abelmoschus manihot* Polysaccharide Fortifies Intestinal Mucus Barrier to Alleviate Intestinal Inflammation by Modulating *Akkermansia muciniphila* Abundance. Acta Pharm. Sin. B.

[68] Kim S., Shin Y.-C., Kim T.-Y., Kim Y., Lee Y.-S., Lee S.-H., Kim M.-N., O E., Kim K.S., Kweon M.-N. (2021). Mucin Degrader *Akkermansia muciniphila* Accelerates Intestinal Stem Cell-Mediated Epithelial Development. Gut Microbes.

[69] Khan S., Waliullah S., Godfrey V., Khan M.A.W., Ramachandran R.A., Cantarel B.L., Behrendt C., Peng L., Hooper L.V., Zaki H. (2020). Dietary Simple Sugars Alter Microbial Ecology in the Gut and Promote Colitis in Mice. Sci. Transl. Med..

[70] Fuhren J., Schwalbe M., Boekhorst J., Rösch C., Schols H.A., Kleerebezem M. (2021). Dietary Calcium Phosphate Strongly Impacts Gut Microbiome Changes Elicited by Inulin and Galacto-Oligosaccharides Consumption. Microbiome.

[71] Pu D., Liang T., Li X., Xing W., Luo L., Ding Y., Wan C., Wu M. (2026). Physicochemical Properties and Molecular Weight-Dependent Gut Microbiota Modulation of Inulin-Type Fructans from *Codonopsis bulleynana*. Carbohydr. Polym..

[72] He Z., Dong H. (2023). The Roles of Short-Chain Fatty Acids Derived from Colonic Bacteria Fermentation of Non-Digestible Carbohydrates and Exogenous Forms in Ameliorating Intestinal Mucosal Immunity of Young Ruminants. Front. Immunol..

[73] Singh V., Lee G., Son H., Koh H., Kim E.S., Unno T., Shin J.-H. (2023). Butyrate Producers, “The Sentinel of Gut”: Their Intestinal Significance with and Beyond Butyrate, and Prospective Use as Microbial Therapeutics. Front. Microbiol..

[74] Lin P.-Y., Stern A., Peng H.-H., Chen J.-H., Yang H.-C. (2022). Redox and Metabolic Regulation of Intestinal Barrier Function and Associated Disorders. Int. J. Mol. Sci..

[75] Kennedy P.M., Milligan L.P. (1978). Effects of Cold Exposure on Digestion, Microbial Synthesis and Nitrogen Transformations in Sheep. Br. J. Nutr..

[76] Xue F., Chen Z., Wang J., Zhao J., Liu F., Zhang C., Liu W., Hao X., Xu S. (2025). Solid-State Fermented *Artemisia argyi* Residue Improves Immune Response and Intestinal Integrity of Broilers Challenged with *Clostridium perfringens*. Poult. Sci..

[77] Song Z.H., Cheng K., Zheng X.C., Ahmad H., Zhang L.L., Wang T. (2018). Effects of Dietary Supplementation with Enzymatically Treated *Artemisia annua* on Growth Performance, Intestinal Morphology, Digestive Enzyme Activities, Immunity, and Antioxidant Capacity of Heat-Stressed Broilers. Poult. Sci..

[78] Wang X., Zhao P., Zhang C., Li C., Ma Y., Huang S. (2024). Effects of Supplemental *Glycyrrhiza* Polysaccharide on Growth Performance and Intestinal Health in Weaned Piglets. Anim. Biotechnol..

[79] Guo S., Ma J., Xing Y., Xu Y., Jin X., Yan S., Shi L., Zhang L., Shi B. (2023). Effects of *Artemisia annua* L. Water Extract on Growth Performance and Intestinal Related Indicators in Broilers. J. Poult. Sci..

[80] Gang G., Gao R., Tong M., Zhang S., Guo S., Jin X., Xing Y., Yan S., Xu Y., Shi B. (2026). Effect of Water Extract of *Artemisia annua* L. on Growth Performance, Blood Biochemical Parameters and Intestinal-Related Indices in Mutton Sheep. Animals.

[81] Bhat A.R., Ishfaq A., Ganai A.M., Beigh Y.A., Sheikh G.G., Masood D. (2017). Effect of *Artemisia absinthium* (Titween) on Nutrient Intake, Digestibility, Nutrient Balance and Blood Biochemical of Sheep. Indian J. Anim. Res..

